# Uniform P-Doped MnMoO_4_ Nanosheets for Enhanced Asymmetric Supercapacitors Performance

**DOI:** 10.3390/molecules29091988

**Published:** 2024-04-26

**Authors:** Yu Liu, Yan Li, Zhuohao Liu, Tao Feng, Huichuan Lin, Gang Li, Kaiying Wang

**Affiliations:** 1 Institute of Energy Innovation, College of Materials Science and Engineering, Taiyuan University of Technology, Taiyuan 030024, China; liuyu0226@link.tyut.edu.cn (Y.L.); liuzhuohao0286@link.tyut.edu.cn (Z.L.); fengtao22657@163.com (T.F.); ligang02@tyut.edu.cn (G.L.); 2 Key Laboratory of Light Field Manipulation and System Integration Applications in Fujian Province, School of Physics and Information Engineering, Minnan Normal University, Zhangzhou 363000, China; lhc1810@mnnu.edu.cn; 3 Department of Microsystems, University of South-Eastern Norway, 3184 Horten, Norway

**Keywords:** asymmetric supercapacitor, MnMoO_4_, nanosheets, phosphorus doping

## Abstract

Manganese molybdate has garnered considerable interest in supercapacitor research owing to its outstanding electrochemical properties and nanostructural stability but still suffers from the common problems of transition metal oxides not being able to reach the theoretical specific capacitance and lower electrical conductivity. Doping phosphorus elements is an effective approach to further enhance the electrochemical characteristics of transition metal oxides. In this study, MnMoO_4_·H_2_O nanosheets were synthesized on nickel foam via a hydrothermal route, and the MnMoO_4_·H_2_O nanosheet structure was successfully doped with a phosphorus element using a gas–solid reaction method. Phosphorus element doping forms phosphorus–metal bonds and oxygen vacancies, thereby increasing the charge storage and conductivity of the electrode material. The specific capacitance value is as high as 2.112 F cm^−2^ (1760 F g^−1^) at 1 mA cm^−2^, which is 3.2 times higher than that of the MnMoO_4_·H_2_O electrode (0.657 F cm^−2^). The P–MnMoO_4_//AC ASC device provides a high energy density of 41.9 Wh kg^−1^ at 666.8 W kg^−1^, with an 84.5% capacity retention after 10,000 charge/discharge cycles. The outstanding performance suggests that P–MnMoO_4_ holds promise as an electrode material for supercapacitors.

## 1. Introduction

The swift growth of the worldwide economy has led to a rise in the extraction and utilization of fossil fuels like oil and coal. Consequently, nonrenewable energy reservoirs are progressively dwindling [1]. With the advancement and application of electrical energy, the imperative lies in creating high-performance electrical energy storage devices to minimize secondary energy wastage [2]. Supercapacitors, positioned between traditional capacitors and batteries, possess a blend of characteristics from both: high capacity, rapid charging and discharging, extended cycle life, and elevated energy density [3,4]. Supercapacitors are primarily categorized into double-layer capacitors (EDLCs) and pseudocapacitors (PCs) based on the charge storage mechanism [5,6]. Selecting the appropriate electrode materials is crucial for the practical implementation of energy storage supercapacitors. Carbon-based materials are commonly employed as electrodes in EDLCs, while transition metal oxides and conducting polymers are frequently utilized as electrode materials for pseudocapacitors. The capacitors with carbon-based electrode materials suffer from low energy storage and poor stability, which conducting polymer layer tends to detach from the substrate [7,8,9]. Therefore, transition metal oxides (TMOs) are favored by researchers because of their generally large theoretical specific capacitance and are often used as electrode materials in energy storage supercapacitors [10].

Transition metal oxides like Fe_3_O_4_, MnO_2_, RuO_2_, NiO, etc. are commonly employed as electrode materials in supercapacitors, but these unit transition metal oxides generally have the disadvantage in their actual specific capacitances being much smaller than the theoretical specific capacitances [11,12,13,14]. Therefore, research workers have focused on binary transition metal oxides, mainly including spinel cobaltates (XCo_2_O_4_, X = Ni, Mn, Zn, etc.) and molybdates (YMoO_4_, Y = Ni, Co, Mn, etc.) [15]. Characterized by the low cost of abundant molybdenum ore resources and multiple oxidation valence states (+3–+6) for easy storage of charge, molybdate is well suited to supercapacitor cathode material [15,16]. Among them, manganese molybdate has good structural stability (compared to cobalt-based molybdates and nickel-based molybdates) due to its special structure and low cohesive energy [16,17]. Manganese molybdate boasts a high theoretical specific capacity (998 mAh g^−1^), stemming from the synergistic effect of the two elements of Mo and Mn (molybdenum ions provide electronic conductivity and manganese ions provide redox activity) [18].

In order to make the actual specific capacitance of manganese molybdate as close as possible to the theoretical value, one approach is to synthesize nanoscale MnMoO_4_ electrodes of a specific micromorphological structure. For instance, Mu et al. synthesized MnMoO_4_·nH_2_O nanosheets on nickel foam using a one-step hydrothermal method, achieving a specific capacitance of 1271 F g^−1^ at a scan rate of 5 mV s^−1^ with 84.5% capacitance retention after 2000 charge/discharge cycles [19]. Doping P, S, and other anions in binary transition metal oxides has been demonstrated to enhance electrical conductivity and promote more extensive oxide reduction reactions, thereby enhancing the charge storage capacity of the electrode materials [20,21,22]. For instance, Meng et al. synthesized uniform P-doped Co–Ni–S nanosheet arrays as binder-free electrodes, exhibiting an ultra–high specific capacitance of 3677 F g^−1^ at 1 A g^−1^ and outstanding cycling stability (approximately 84% capacitance retention after 10,000 charge/discharge cycles) [21].

The electronic arrangement of the element phosphorus leads to multivalent, metal-like properties and better electrical conductivity of transition metal phosphides compared to transition metal oxides, due to the relatively narrow gap between their conduction and valence bands, and the excellent electrical conductivity is very favorable for electrochemical energy storage processes [23]. Transition metal phosphides can be regarded as phosphorus elements doped into transition metals and their oxides [24]. It is the gas–solid reaction method that the phosphine gas involved in the phosphorylation reaction makes the phosphorus element doped into the metal oxide. The advantage of this method is that the morphological structure of the phosphated product remains essentially the same as that of the precursor. However, because phosphine is highly toxic, the gas–solid reaction is generally chosen to decompose hypophosphite into phosphine gas by heating, which then participates in the phosphorylation reaction [25,26].

In this research, MnMoO_4_·H_2_O nanosheets were initially synthesized directly on nickel foam using the hydrothermal method. Then, the prepared MnMoO_4_·H_2_O nanosheets were subjected to phosphorus doping in a tube furnace using a gas–solid reaction method. Sodium hypophosphite was used as the phosphorus source, and the experimental parameters of the phosphorus source content, phosphorylation reaction temperature, and reaction time were optimized for phosphorylation.

## 2. Results and Discussion

### 2.1. Structure and Morphology Analysis

**Figure 1:**
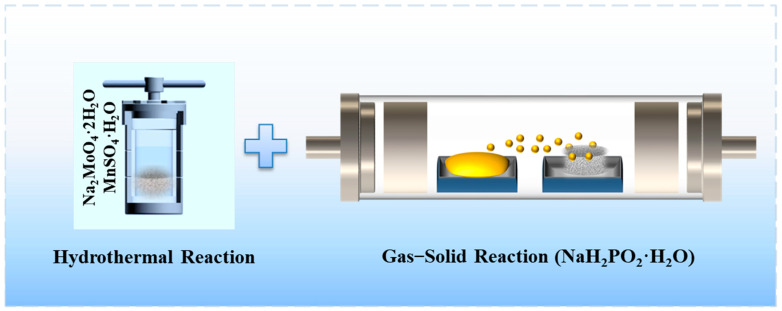
Diagram illustrating the preparation of P–MnMoO_4_/NF.

Figure 1 illustrates the preparation process of phosphorus-doped MnMoO_4_ nanomaterials on NF. With the clean nickel foam immersed in a mixed solution of MnSO_4_⋅H_2_O and Na_2_MoO_4_⋅2H_2_O the first step of the hydrothermal reaction at 150 °C for 8 h yielded a nanosheet array of MnMoO_4_⋅H_2_O grown on the nickel foam. The nickel foam that has gone through the first hydrothermal process and the sodium hypophosphite powder were placed into a tubular furnace side by side. Phosphorus element doping was achieved using the gas–solid reaction method, with NaH_2_PO_2_⋅H_2_O positioned upstream in an argon atmosphere and MnMoO_4_⋅H_2_O/NF positioned downstream.

**Figure 2:**
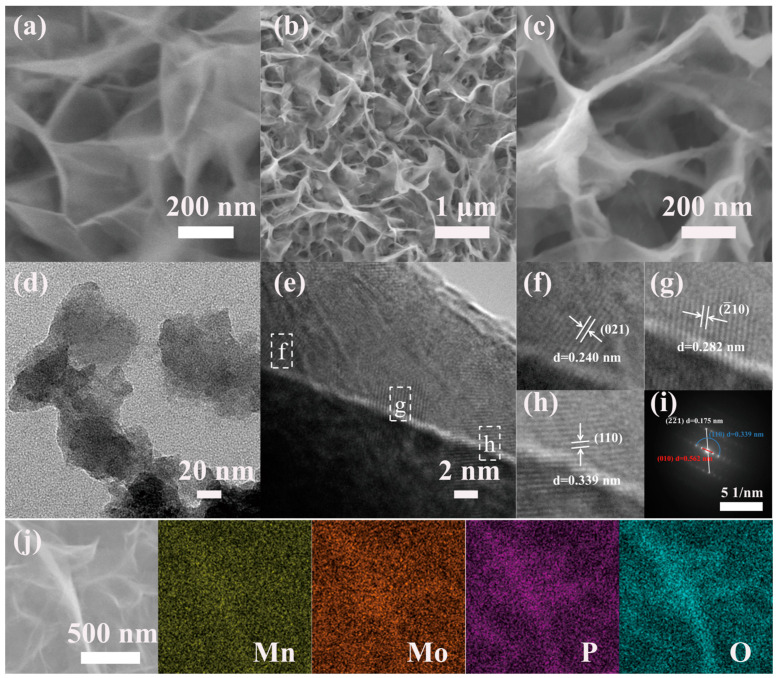
SEM images of (**a**) MnMoO_4_·H_2_O and (**b**,**c**) P–MnMoO_4_; (**d**) TEM and (**e**–**h**) HRTEM images of P-MnMoO_4_ and (**i**) the corresponding SAED pattern; and (**j**) EDS mapping of P–MnMoO_4_.

The morphologies of the MnMoO_4_·H_2_O and P–MnMoO_4_ nanosheets were analyzed by SEM. Before phosphorylation, manganese molybdate presents as a dense, uniform, vertically aligned array of nanosheets on the surface of NF. The cores of MnMoO_4_·H_2_O nanosheets present a regular morphology and crosslinking structure without aggregation (Figure 2a). This architecture minimizes the electrode material’s inactive volume and enhances the electron conduction efficiency during electrochemical processes. Figure 2b shows that the phosphorus-doped manganese molybdate sample is vertically interconnected, and the addition of phosphorus does not change the original morphological structure of the samples. The skeleton of the nickel foam substrate is covered by a layer of uniformly dense nanosheets. Possibly due to the distribution at the edges of the nickel foam skeleton, some aggregates and nanoflowers appear, which have little effect on the overall morphology, and a small number of nanoflowers can increase the specific surface area and improve the electrochemical properties (Figure S1). However, the surface of the nanosheets becomes coarse, and the surface is covered with separated particles producing a large number of marginal sites of small size effects (Figure 2c). These alterations lead to an increased specific surface area of the P–MnMoO_4_ nanosheets electrode material, enhancing the electrical contact with the electrolyte. Additionally, the incorporation of phosphorus elements enhances the overall electrical conductivity and promotes electrochemical activity.

The nanosheet structure of the P–MnMoO_4_ nanomaterial was analyzed using TEM images. Figure 2d shows the TEM image of P–MnMoO_4_, revealing a distinct nanosheet structure. Figure 2e shows the HRTEM image of P–MnMoO_4_ with clear lattice fringes. The lattice distances of 0.240 nm, 0.282 nm, and 0.339 nm depicted in Figure 2f–h correspond to the (021), ($\bar{2}10$), and (110) planes of the MnMoO_4_⋅H_2_O phase, respectively. Figure 2i shows the SAED pattern of P–MnMoO_4_, indicating its polycrystalline nature with distinct spots and rings. The SAED pattern matches the ($\bar{2}\bar{2}$1), (110), and (010) planes of MnMoO_4_⋅H_2_O, indicating that a small amount of phosphorus doping does not affect the MnMoO_4_⋅H_2_O nanosheet substrate. To ascertain the elemental composition of the experimental samples, EDS scans were conducted on the doped samples. Figure 2j shows the P–MnMoO_4_ scanning the EDS diagram at the magnification surface. Mn, Mo, O, and P are evenly dispersed across the nickel foam’s surface. The successful doping of phosphorus atoms into manganese molybdate was demonstrated.

**Figure 3:**
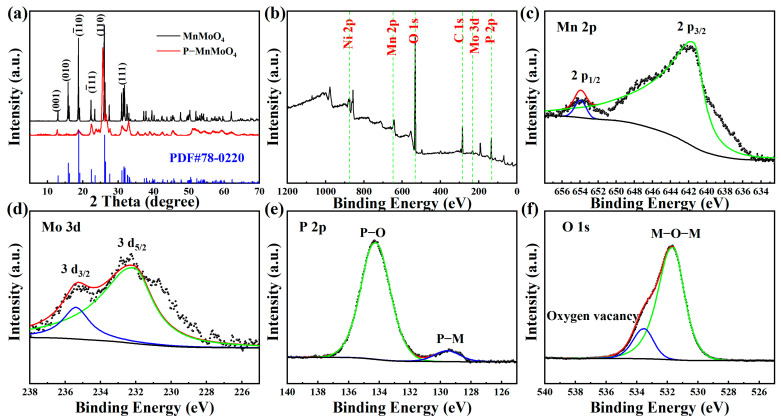
(**a**) XRD patters of MnMoO_4_·H_2_O and P–MnMoO_4_, and (**b**) the XPS survey spectrum of the P–MnMoO_4_, (**c**) Mn 2p, (**d**) Mo 3d, (**e**) P 2p, and (**f**) O 1s spectrum.

To examine the crystal structure and composition of the samples, XRD analysis was conducted on the prepared MnMoO_4_·H_2_O and P–MnMoO_4_ nanosheets, as depicted in Figure 3a. Because the X-ray diffraction peak of nickel is rather strong and the amount of MnMoO_4_·H_2_O grown in situ is low, the active material was first scraped off from the nickel foam, and the scraped nickel monomers were absorbed with a magnet for XRD testing. The diffraction peaks of MnMoO_4_·H_2_O grown in situ by the hydrothermal method are consistent with the standard triclinic MnMoO_4_·H_2_O (JCPDS card No.78–0220) [27]. Among them, the characteristic peaks with 2θ of 12.92°, 15.94°, 18.79°, 26.28°, and 31.98° correspond to the (001), (010), ($\bar{1}10$), (110), and (111) crystal plane diffractions of MnMoO_4_·H_2_O, respectively. Meanwhile, the high and fine diffraction peaks of MnMoO_4_·H_2_O indicate better crystallinity.

The diffraction pattern of P–MnMoO_4_ did not change significantly, indicating that only a small amount of phosphorus was doped during the gas–solid reaction. The diffraction peaks of P–MnMoO_4_ at 12.71°, 18.74°, 25.65°, and 31.87° correspond to the (001), ($\bar{1}10$), (110), and (111) crystal plane diffractions of MnMoO_4_·H_2_O, respectively. It shows that the structure of manganese molybdate is not changed after phosphorylation, which is consistent with the SEM results. The synthesized material is uniform in composition rather than being composite. The low and broad diffraction peaks of P–MnMoO_4_ compared to those of MnMoO_4_·H_2_O indicate that poorer crystallinity was obtained. This difference may be caused by P doping, in which the larger radius P elements partially replace the original position of O, which may lead to a slight change in the crystallinity [28], and no additional diffraction peaks appeared, indicating that the doping of the P element did not change the original crystal structure.

To gain a more thorough insight into the elemental composition and chemical states of P–MnMoO_4_ nanosheets, XPS analysis was performed. The XPS (Figure 3b) of P–MnMoO_4_ demonstrated the existence of Mo, Mn, P, and O elements. The binding energy peaks at 653.73 eV and 641.40 eV are attributed to Mn 2p_1/2_ and Mn 2p_3/2_, respectively (Figure 3c). The energy gap between these two peaks is 12.33 eV, suggesting the presence of Mn^2+^ [29,30]. In Figure 3d, the binding energy peaks at 235.45 eV and 231.74 eV are attributed to Mo 3d_3/2_ and Mo 3d_5/2_, respectively. The energy gap between these two peaks is 3.71 eV, suggesting the presence of Mo^6+^ [31,32]. In Figure 3e, the P 2p core energy level spectrum reveals two peaks with binding energies of 134.14 eV and 129.48 eV, corresponding to the P–O bond (phosphide signal peak) and the phosphorus–metal bond, respectively [21,33]. The binding energy peaks at 533.25 eV and 531.60 eV in Figure 3f correspond to the characteristic peaks of the oxygen vacancies and metal–oxygen bonds, respectively [20,34]. There was no significant change observed in the chemical state in the P 2p spectra, suggesting the structural stability of the material. These findings further confirm the successful doping of elemental P into the MnMoO_4_ nanosheets, which are also indicated by the results of the XRD test described previously.

### 2.2. Electrochemical Characterizations

Through prior research experience, the optimal hydrothermal reaction time and temperature conditions were determined for the preparation of the precursor MnMoO_4_·H_2_O nanosheets using the hydrothermal method. The experimental conditions of 150 °C and 8 h are used to generate MnMoO_4_·H_2_O nanosheets of favorable microscopic morphology and pore size for good contact between the electrolyte and active material [20,35]. In the gas–solid reaction method, the thermal decomposition of NaH_2_PO_2_·H_2_O produces PH_3_ gas and water vapor present in the tube furnace. Driven by argon, PH_3_ gas moves to the surface of manganese molybdate and reacts with it to form phosphoric acid. A small amount of phosphoric acid is gradually “acid dissociated” in the presence of water vapor to produce ${HPO}_{4}^{2-}$ and ${H_{2}PO}_{4}^{-}$ in turn. Then, ${H_{2}PO}_{4}^{-}$ and ${OH}^{-}$ undergo ion exchange on the surface of MnMoO_4−4x_, ${OH}^{-}$ diffuses outward, and ${H_{2}PO}_{4}^{-}$ penetrates inward slowly to realize the phosphorus doping. The specific reaction equations are as follows:

NaH_2_PO_2_·H_2_O→PH_3_↑ + Na_2_HPO_4_ + H_2_O↑ (1)

MnMoO_4_ + xPH_3_ = MnMoO_4−4x_ + xH_3_PO_4_ (2)

(3) $$H_{2}O+{PO}_{4}^{3-}={HPO}_{4}^{2-}+{OH}^{-}$$

(4) $${HPO}_{4}^{2-}+H_{2}O={H_{2}PO}_{4}^{-}+{OH}^{-}$$

**Figure 4:**
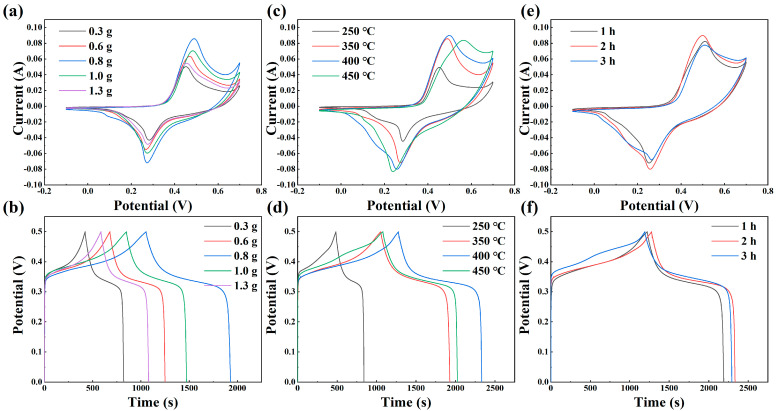
Comparison of the P–MnMoO_4_ electrode under different experimental conditions: (**a**,**b**) CV curves at 20 mV s^−1^ and GCD curves at 1 mA cm^−2^ for different phosphorus source contents; (**c**,**d**) phosphorylation temperatures; (**e**,**f**) phosphorylation times.

In order to determine the optimal phosphorylation reaction conditions, three parameters were studied in terms of the amount of phosphorus source, phosphorylation temperature, and phosphorylation time, respectively. Subsequently, CV and GCD tests were performed to evaluate the impact of these parameters on the electrochemical properties resulting from the phosphorylation reaction. Figure 4a depicts the CV curves of the phosphorylated manganese molybdate electrode at 20 mV s^−1^ for varying phosphorus source quantities: 0.3 g, 0.6 g, 0.8 g, 1.0 g, and 1.3 g. Observably, the curve corresponding to a hypophosphite quantity of 0.8 g covers a larger enclosed area and demonstrates a higher capacitance area ratio. The mass ratio of the precursor to phosphorus source ranges from 1:10 to 1:40 or even higher [36,37]. From the preliminary experiments, the electrochemical properties of P–MnMoO_4_/NF generated by the gas phase reaction were not significantly improved when the content of the phosphorus source (NaH_2_PO_2_) was lower than 0.3 g. The electrochemical properties of P–MnMoO_4_/NF generated by the gas phase reaction were not significantly improved. If the content of the phosphorus source is too much and too high, it may lead to the accumulation of the phosphorus source (NaH_2_PO_2_) before the decomposition reaction, which leads to the ineffective improvement of the electrochemical performance and, at the same time, causes a large amount of phosphorus resources to be wasted. Figure 4b illustrates the GCD curves of the electrodes (0–0.5 V) following the phosphorylation of manganese molybdate with different phosphorus source amounts at 1 mA cm^−2^. The amount of hypophosphite is 0.8 g for the longest discharge time and higher charging and discharging plateau voltage, so it is determined that 0.8 g is the optimal amount of sodium hypophosphite for the phosphorus source.

The effect of the phosphorylation temperature on the experimental results was studied based on the phosphorus source content of 0.8 g and the temperatures of 250 °C, 350 °C, 400 °C, and 450 °C, respectively. Figure 4c illustrates the CV plots of the three samples at different temperatures at a 20 mV s^−1^ scan rate. It can be seen that P–MnMoO_4_ are pseudocapacitor materials at three different temperatures, but there is little difference in the wrapping area of the CV curves at 400 °C and 450 °C. Constant current charge and discharge are tested and shown in Figure 4d, in which the GCD curve of 400 °C has the longest discharge time, so the optimal phosphorylation reaction temperature is determined to be 400 °C. The experiments to determine the phosphorylation time were conducted under the condition of 0.8 g sodium hypophosphite and a reaction temperature of 400 °C. The reaction durations chosen were 1 h, 2 h, and 3 h, respectively. CV and GCD tests were conducted at identical scan rates and current densities, respectively (Figure 4e,f). The area enclosed by the CV curves is difficult to directly assess, indicating that the phosphorylation time has minimal impact on the electrochemical performance. The GCD curves were measured to quantitatively analyze the respective specific capacitance, and the optimal phosphorylation reaction time of 2 h was subsequently determined. In summary, the ideal parameters for the phosphorylation experiment were 0.8 g of sodium hypophosphite, a reaction temperature of 400 °C, and a reaction time of 2 h. The electrochemical performance of P–MnMoO_4_ and MnMoO_4_·H_2_O prepared under the optimal experimental conditions was compared.

We compared the electrochemical performance of the P–MnMoO_4_ electrode (0.8 g, 400 °C, 2 h) prepared with the optimal parameters separately with that of the MnMoO_4_·H_2_O electrode. Detailed CV and GCD curves for the MnMoO_4_·H_2_O and P–MnMoO_4_ electrode materials at different scan rates and current densities are shown in Figures S2 and S3. The area specific capacitances of the MnMoO_4_·H_2_O electrodes are 0.657, 0.634, 0.605, 0.550, 0.505, 0.451, and 0.400 F cm^−2^ at current densities of 1, 2, 3, 5, 10, 15, and 20 mA cm^−2^. The CV curves of the P–MnMoO_4_ electrode (0.8 g, 400 °C, 2 h) at various scanning rates exhibited minimal change in curve morphology, suggesting excellent reversibility of the electrode.

**Figure 5:**
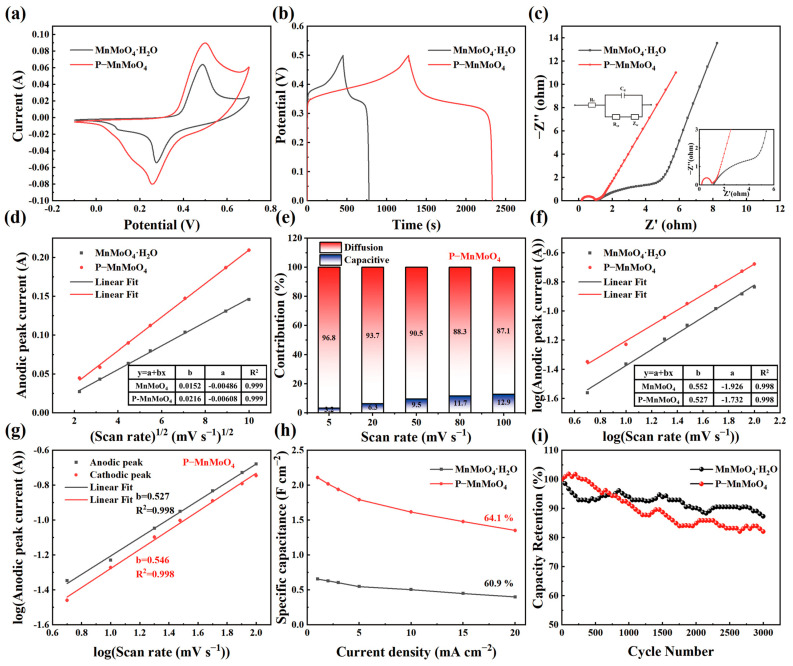
Comparison of MnMoO_4_·H_2_O and P–MnMoO_4_: (**a**) CV curves at 20 mV s^−1^; (**b**) GCD curves at 1 mA cm^−2^; (**c**) Nyquist plots (insets show the corresponding high-magnified EIS and equivalent circuit); (**d**) relationship between the peak anode current and square root of the sweep rate; (**e**) proportions of capacitive and diffusion-controlled contributions at various scan rates of the P–MnMoO_4_ electrode; (**f**) relationship between log (|i|) and log (v); (**g**) the log (|i|) versus log (v) plots of the cathodic and anodic peak current responses of the P–MnMoO_4_ electrode; (**h**) rate capability; (**i**) stability test.

Figure 5a depicts the CV curves of the P–MnMoO_4_ and MnMoO_4_·H_2_O electrodes within the potential range of −0.1 to 0.7 V at a scanning rate of 20 mV s^−1^. The enclosed area of the CV curve for the P–MnMoO_4_ electrode exceeds that of the MnMoO_4_·H_2_O electrode, indicating that P–MnMoO_4_ can store more charge and has better electrochemical performance, mainly attributed to the addition of phosphorus elements. Both CV curves exhibit a pair of well-defined redox peaks, indicative of Faraday reactions associated with electrochemical capacitance. In Figure 5b, the GCD curves of the P–MnMoO_4_ and MnMoO_4_·H_2_O electrodes are shown, measured at 1 mA cm^−2^. The discharge time of the P–MnMoO_4_ electrode (1054 s) notably surpasses that of the MnMoO_4_·H_2_O electrode (327 s). At 1 mA cm^−2^, the specific capacitance of P–MnMoO_4_ is 2.112 F cm^−2^, approximately 3.2 times greater than that of the MnMoO_4_·H_2_O electrode (0.657 F cm^−2^). The two electrodes both exhibit charge/discharge plateaus, indicating the pseudocapacitive characteristics of the active material. In Figure 5c, Nyquist plots of the P–MnMoO_4_ and MnMoO_4_·H_2_O electrodes are displayed, with the inset illustrating the equivalent circuit diagram. Since both materials are grown on nickel foam, the contact resistance is minimal and manifests at the intersection of the impedance curve with the horizontal axis. The radius of the curvature of P–MnMoO_4_ in the high-frequency region is smaller than that of MnMoO_4_·H_2_O, indicating a reduced charge transfer resistance. In the low-frequency region, a linear trend with a slope close to 1 represents the Warburg impedance, reflecting the efficiency of electrolyte ion transfer at the electrode surface and in solution. The findings indicate that the internal resistance (R_s_ = 0.198 Ω) and charge transfer resistance (R_ct_ = 0.735 Ω) of P–MnMoO_4_ are lower than those of MnMoO_4_·H_2_O (internal resistance (R_s_ = 1.121 Ω) and charge transfer resistance (R_ct_ = 5.398 Ω)), attributed to the incorporation of phosphorus to enhance the overall conductivity of the electrode material.

Figure 5d shows that the corresponding currents of the redox peaks of the P–MnMoO_4_ and MnMoO_4_·H_2_O electrodes are roughly linear with the one-half order of the sweep speed. It shows that the energy storage of the P–MnMoO_4_ and MnMoO_4_·H_2_O electrodes is mainly carried out by the redox reaction inside the electrode material, not only by the surface redox reaction [38]. The P–MnMoO_4_ electrode has a larger slope of the fitted line (b = 0.0216), indicating a high ion migration rate. Indirectly, it is proven that the phosphorus element is doped into the interior of MnMoO_4_·H_2_O and participates in the redox reaction, possibly forming oxygen vacancies or phosphorus atoms replacing oxygen atoms. Figure 5e shows the contribution rates of the surface-controlled and diffusion-controlled capabilities of the P–MnMoO_4_ electrode at different scan rates. As the scan rates increase, the surface-controlled capabilities become more prominent due to the suppression of ion diffusion [39]. However, at 100 mV s^−1^, the diffusion-controlled reaction capacitance remains dominant at 87.3%, indicating that the fast redox reaction process of the P–MnMoO_4_ electrode in electrochemical reactions is less affected by the scan rate, corresponding to the high ion migration rate. Figure 5f illustrates the fitting line of log (i) versus log (v) collected from the CV curve of various electrodes. The constant of the P–MnMoO_4_ electrode is 0.527, closer to 0.5, revealing that the P–MnMoO_4_ electrode is a typical diffusion-controlled Faraday reaction. In Figure 5g, the fitting b values of the oxidation and reduction peaks of the P–MnMoO_4_ electrode are displayed, both approaching 0.5. This suggests excellent reversibility in the redox reaction of the P–MnMoO_4_ electrode, facilitating rapid and reversible electron transfer at the interface between the electrode material and the electrolyte.

Area-specific capacitances for the P–MnMoO_4_ and MnMoO_4_·H_2_O electrodes were computed from the GCD curves at various current densities (Figure 5h). As the current density increased, the specific capacitance of both the P–MnMoO_4_ and MnMoO_4_·H_2_O electrodes decreased. Due to the rapid decrease in the charge/discharge time at higher charge/discharge rates, the movement of ions is restricted and the ions cannot reach the interior of the electrode material in a short time, and the redox and ion intercalation reactions occur incompletely [40]. As the current density increased from 1 mA cm^−2^ to 20 mA cm^−2^, the specific capacitance retention of the P–MnMoO_4_ electrode was 64.1%, which was higher than that of the MnMoO_4_·H_2_O electrode (60.9%). The P–MnMoO_4_ and MnMoO_4_·H_2_O electrodes were charged and discharged 3000 times at 5 mA cm^−2^ (Figure 5i). After 3000 charge/discharge cycles, the specific capacitance retention rate of P–MnMoO_4_ was 82.1%, slightly lower than that of the MnMoO_4_·H_2_O electrode (87.3%). The nanosheet morphology of the P–MnMoO_4_ electrode material does not change after long-term cycling (Figure S4). The decrease in capacity retention after multiple charge/discharge cycles is mainly due to the possible slight exfoliation of the nanosheet structure of the electrode material and the change in electrolyte concentration during long cycling. Nonetheless, the specific capacity after cycling of the P–MnMoO_4_ electrode remains higher than the specific capacity before cycling of the MnMoO_4_·H_2_O electrode. Overall, the P–MnMoO_4_ electrode material still demonstrates favorable cycling stability.

### 2.3. P–MnMoO_4_//AC ASC Testing

**Figure 6:**
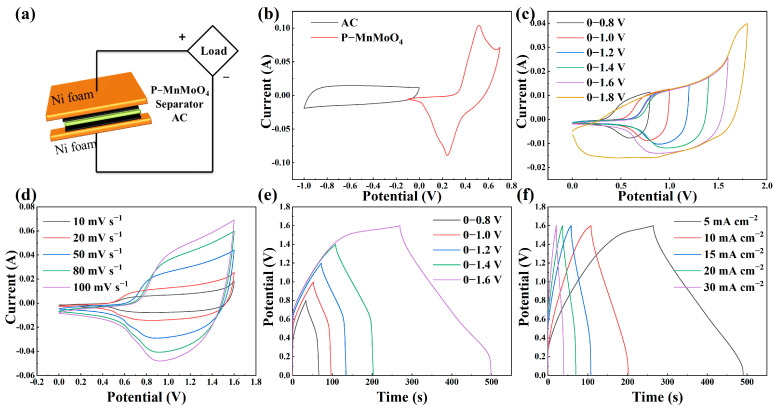
(**a**) Schematic representation of the assembled hybrid P–MnMoO_4_//AC supercapacitor; (**b**) CV curves for the positive and negative electrodes at 20 mV s^−1^; (**c**) CV curves at different voltage windows (20 mV s^−1^); (**d**) CV curves, (**e**) GCD curves at different voltage windows (5 mA cm^−2^); (**f**) GCD curves.

To evaluate P–MnMoO_4_’s practical utility, we constructed an asymmetric supercapacitor device with P–MnMoO_4_ serving as the positive electrode and activated carbon (AC) as the negative electrode, 2 M KOH as the electrolyte, and cellulose paper as the diaphragm (Figure 6a). Detailed CV and GCD curves for commercial activated carbon (AC) anode electrode materials are given in Figure S5, and the CV curves are quasi-rectangular in shape, which is a double electric layer capacitance characteristic. In the three-electrode test regime, Figure 6b shows the CV curves measured at 20 mV s^−1^ for P–MnMoO_4_ and activated carbon, respectively. The absence of overlap between the individual CV curve regions of the positive and negative electrodes within the potential window confirms the precise alignment of the two electrodes during the assembly of the asymmetric supercapacitor [41]. To establish the voltage window of the device, we expanded the voltage range of the CV curve from 0–0.8 V to 0–1.8 V at a scan rate of 20 mV s^−1^ (Figure 6c). There was no significant polarization in the 0–1.6 V range, and the P–MnMoO_4_//AC device was CV tested at scan rates of 10 to 100 mV s^−1^ during this voltage window (Figure 6d). As the scan rate increases, all CV curve shapes do not change due to the increase in scan speed, and they are irregularly rectangular in shape. Both pseudocapacitors and double-layer capacitors contribute to this asymmetric supercapacitor device [42]. Figure 6e shows the GCD curve that indicates the maximum voltage window, which is obtained by incrementing the voltage from 0 to 0.8 V with a step of 0.2 V at 5 mA cm^−2^. If the GCD test is performed above the 1.6 V voltage window, it will cause the device to remain in the charging state rather than be discharged, and a higher voltage window will not be achieved. The CV and GCD tests incremented the voltage window, and the final test results were consistent, identifying the device voltage window as 0–1.6 V. Figure 6f shows the variation of the GCD curve of P–MnMoO_4_//AC as the current density increases from 5 mA cm^−2^ to 30 mA cm^−2^ within the voltage range of 0–1.6 V. The symmetrical shapes of the CV and GCD curves of the devices tested at different scanning speeds and current densities indicate that the two electrode materials are well matched and have excellent charge/discharge reversibility [38].

**Figure 7:**
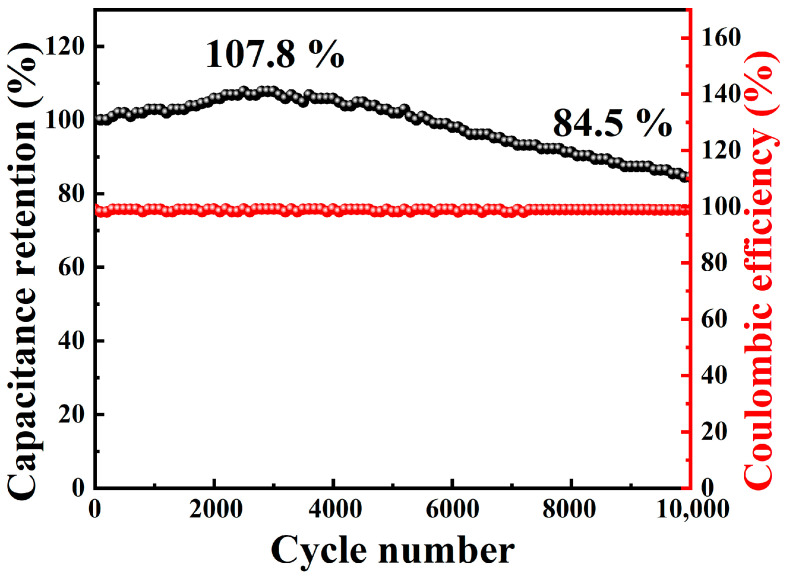
Cycling performance of the P–MnMoO_4_//AC ASC device.

The power and energy density of the P–MnMoO_4_//AC ASC device can be computed using Equations (7) and (8). The energy density of P–MnMoO_4_//AC was 41.9, 34.8, 27.6, 25.3, and 21.2 Wh kg^−1^ for power densities of 666.8, 1348.8, 2015.4, 2751.7, and 4128.3 W kg^−1^, respectively. The capacity retention of the P–MnMoO_4_//AC ASC device was 84.5% after 10,000 cycles, and the Coulombic efficiency remained nearly 100% throughout each charge/discharge cycle, with the initial increase in capacity during the cycling period likely attributable to the activation process of the electrode material (Figure 7). When compared to other asymmetric supercapacitors comprising MnMoO_4_ material and activated carbon, the P–MnMoO_4_//AC supercapacitor demonstrates superior energy density at equivalent power densities and exhibits outstanding cycling stability (Table S1). The results imply the possibility of practical applications of phosphorus-doped manganese molybdate in energy storage devices.

## 3. Materials and Methods

### 3.1. Chemicals and Materials

The reagents included Na_2_MoO_4_·2H_2_O, MnSO_4_·H_2_O, NaH_2_PO_2_·H_2_O, anhydrous ethanol, KOH, HCl, CH_3_COCH_3_, NF, commercial active carbon, acetylene black, polyvinylidene fluoride (PVDF), and N–methyl pyrrolidone, purchased from Sinopharm Chemical Reagents Co. Ltd. (Shanghai, China). The 1 mm thick Ni foams were trimmed into pieces measuring 1 cm × 1.5 cm for ease of handling. Subsequently, they were immersed in 1 M HCl solution and acetone for ultrasonic cleaning for 15 min to eliminate NiO and organic contaminants from the surface. Afterwards, the pretreated Ni foams underwent thorough rinsing with deionized water and ethanol before being vacuum-dried at 60 °C for 12 h. All reagents utilized were of analytical grade and necessitated no further purification.

### 3.2. Synthesis of MnMoO_4_·H_2_O Precursors and P–MnMoO_4_

In a typical synthesis, 2 mmol of Na_2_MoO_4_·2H_2_O and 2 mmol of MnSO_4_·H_2_O were dissolved separately in 40 mL of deionized water. The mixed solution and clean Ni foam were then transferred into a 100 mL stainless steel autoclave and placed in a blast-drying oven at 150 °C for 8 h. After the reaction was completed, the samples were cooled to room temperature, gently rinsed with deionized water to prevent detachment of the grown MnMoO_4_·H_2_O from the nickel foam, and subsequently dried at 60 °C for 12 h.

The doping of the phosphorus element was achieved using a gas–solid reaction method. MnMoO_4_·H_2_O/NF and NaH_2_PO_2_·H_2_O were placed in the porcelain boat, with NaH_2_PO_2_·H_2_O positioned upstream and MnMoO_4_·H_2_O/NF downstream. Then, it was placed in a tube furnace, heated with argon gas to a certain temperature for a certain period of time, and then cooled to room temperature to obtain P–MnMoO_4_/NF. In this paper, we proposed the optimization of the experimental parameters of the phosphorus source (0.3 g, 0.6 g, 0.8 g, 1.0 g, and 1.3 g); phosphating temperature (250 °C, 350 °C, 400 °C, and 450 °C); and phosphating time (1 h, 2 h, and 3 h) to generate P–MnMoO_4_. The mass of active material on NF of the MnMoO_4_·H_2_O and P–MnMoO_4_ (0.8 g, 400 °C, 2 h) samples was about 1 mg cm^−2^ and 1.2 mg cm^−2^, respectively, measured by an electronic balance.

### 3.3. P–MnMoO_4_//AC Asymmetric Supercapacitor Assembly

Activated carbon (AC), conductive carbon black, and polyvinylidene fluoride (PVDF) were mixed in a mass ratio of 8:1:1 and combined with an appropriate amount of N–methylpyrrolidone. The mixture was stirred into a paste at room temperature and then uniformly applied to clean Ni foam to prepare the negative electrode of the device. This asymmetric device was assembled at room temperature and in air and used for the two-electrode test. The amount of AC required was calculated according to Equation (5) [43]:

(5) $$\frac{m^{+}}{m^{-}}=\frac{C^{-}\times \Delta V^{-}}{C^{+}\times \Delta V^{+}}$$

where m (g), C (F cm^−2^), and ΔV (V) represent the mass of electrode material, specific capacitance, and potential window, respectively.

### 3.4. Characterization of Materials

The nanostructured morphologies of the samples were examined using a scanning electron microscope (SEM, ZEISS Gemini 300, Jena, Germany). Elemental mapping imaging was performed using energy dispersive X-ray spectroscopy (EDS, Horiba EMAX Energy, EX-350, Kyoto, Japan). Transmission electron microscopy (TEM), high-resolution transmission electron microscopy (HRTEM), and selected area electron diffraction (SAED) images were obtained with a FEI-TALOS-F200X (Thermo Fisher Scientific, Waltham, MA, USA). The crystal structure of the samples was analyzed using an X-ray powder diffractometer (XRD, Empyrean, Malvern Panalytical B.V, Almelo, The Netherlands) with graphite monochromatic Cu Kα irradiation. The chemical compositions of the nanocomposites were analyzed using X-ray photoelectron spectroscopy (XPS, American Thermo Fisher Scientific K-Alpha, USA).

### 3.5. Electrochemical Measurements

All electrochemical measurements were performed using an electrochemical workstation (CHI 660D). Samples of MnMoO_4_·H_2_O and P–MnMoO_4_ electrode materials prepared on Ni foam were directly used as working electrodes for the three-electrode test, and Pt net and Hg/HgO electrodes were used as counter and reference electrodes in 2 M KOH aqueous electrolytes. The specific capacitance of the single and full electrode devices were calculated using Equation (6) [44]:

(6) $$C_{s}=\frac{I\times \Delta t}{m\times \Delta V}$$

where I (A) is the discharge current, Δt (s) is the discharge time, m (g) is the mass loading of the active material, ΔV is the operating voltage, and $C_{s}$ (F g^−1^) is the mass ratio capacitance. When its m (g) is replaced with the effective area of the electrode, Equation (6) can be used for the calculation of the area ratio capacitance (F cm^−2^).

To calculate the energy and power density of the asymmetric supercapacitor, the following Equations (7) and (8) were used [45]:

(7) $$E=\frac{1}{7.2}CV^{2}$$

(8) $$P=\frac{3600E}{\Delta t}$$

where E (Wh kg^−1^) is the energy density, P (W kg^−1^) stands for the power density, C (F g^−1^) is the specific capacitance, V(V) is the operating voltage window, and Δt (s) is the discharge time.

## 4. Conclusions

In this research, MnMoO_4_·H_2_O nanosheets were initially synthesized on nickel foam via a hydrothermal approach, followed by the introduction of phosphorus into the MnMoO_4_·H_2_O nanosheets using a gas–solid reaction method. The experimental parameters of the phosphorus source content, reaction temperature, and reaction duration were optimized for phosphorylation. The phosphorylated manganese molybdate nanosheets were characterized and electrochemically measured. The P–MnMoO_4_ of the preferred electrochemical properties were achieved when the phosphorus source content was 0.8 g, the heating temperature was 400 °C, and the heating time was 2 h. At l mA cm^−2^, the specific capacitance of P–MnMoO_4_ was 2.112 F cm^−2^, approximately 3.2 times greater than that of the MnMoO_4_·H_2_O electrode. Following 3000 charge/discharge cycles at 5 mA cm^−2^, the specific capacitance of P–MnMoO_4_ remained at approximately 82.1% of its initial value. Phosphorus doping enhances the charge storage, conductivity, and ion migration rate of MnMoO_4_ while preserving the nanosheet morphology of MnMoO_4_. P–MnMoO_4_//AC devices provide a high energy density of 41.9 Wh kg^−1^ at a power density of 666.8 W kg^−1^, with 84.5% capacity retention after 10,000 charge/discharge cycles. This work shows that the P–MnMoO_4_ material is a potential electrode material with extensive applications in building high-performance energy storage devices.

## Data Availability

The data presented in this study are available in the article.

## Supplementary Materials

The following Supplementary Materials can be downloaded at https://www.mdpi.com/article/10.3390/molecules29091988/s1: Figure S1. SEM images of (a,b) NF, (c,d) MnMoO_4_·H_2_O, and (e,f) P–MnMoO_4_; Figure S2. (a) CV curve of MnMoO_4_·H_2_O; (b) GCD curve; Figure S3. (a) CV curve of P–MnMoO_4_; (b) GCD curve; Figure S4. (a–c) SEM images of the P–MnMoO_4_ electrode material after charge/discharge cycles; Figure S5. (a) CV curve of activated carbon; (b) GCD curve; Table S1. Performance comparison of the hybrid supercapacitor based on the MnMoO_4_ electrode material with other reported devices. Refs. [46,47,48,49,50,51] are cited in the Supplementary Materials.
