# Supplementary material for: Uniform P-Doped MnMoO4 Nanosheets for Enhanced Asymmetric Supercapacitors Performance
Source: Molecules. 2024 Apr 26;29(9):1988. doi: 10.3390/molecules29091988 (PMC11085725; doi:10.3390/molecules29091988)
Supplement: Supplementary file 1 [file molecules-29-01988-s001.zip › molecules-2969823-supplementary.pdf]

Uniform P−Doped $MnMoO_4$ Nanosheets for Enhanced Asymmetric Supercapacitors Performance

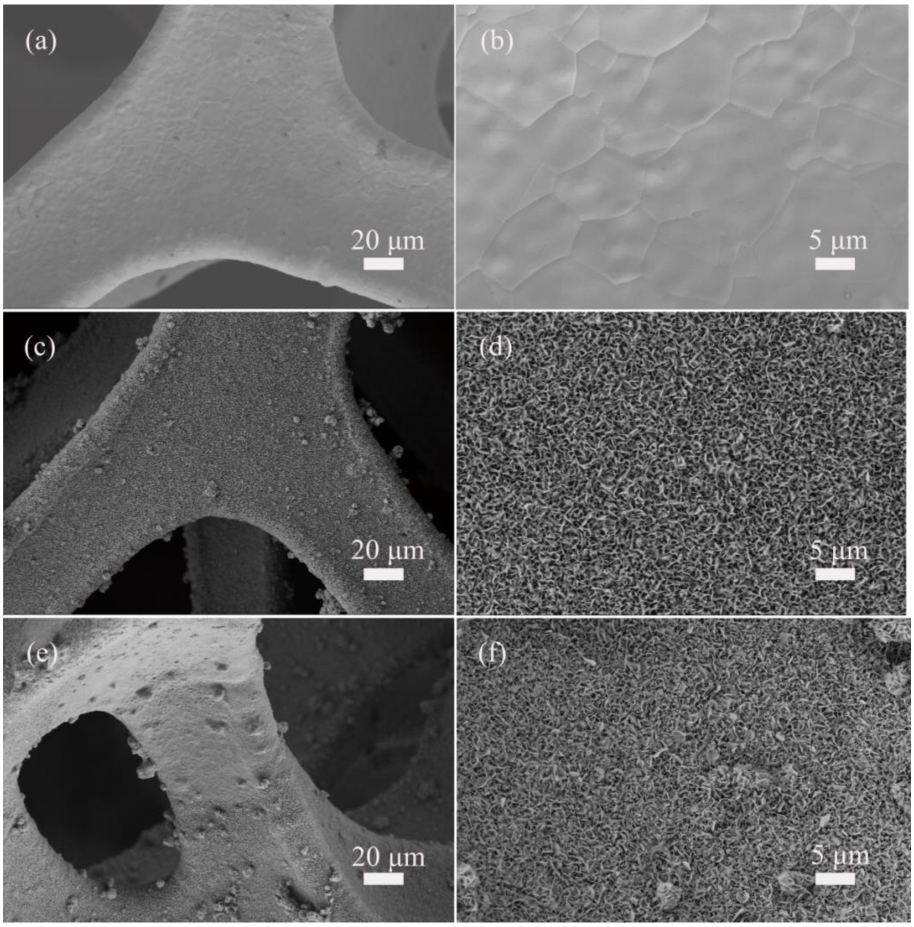

Figure S1. SEM images of (**a, b**) NF (**c, d**) $MnMoO_4{\cdot}H_2O$ and (**e, f**) P−$MnMoO_4$

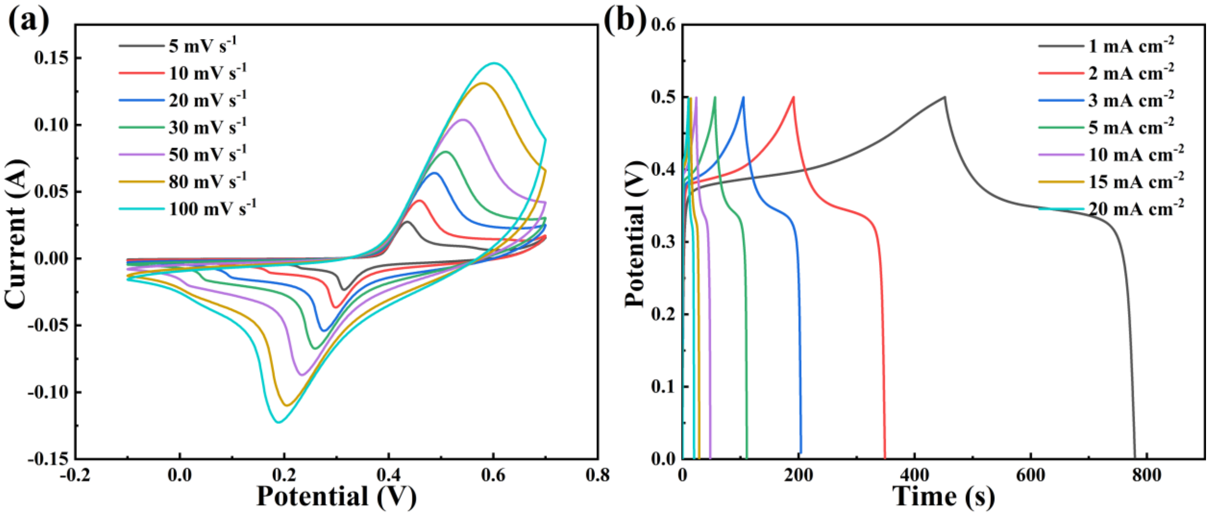

Figure S2. (**a**) CV curve of $MnMoO_4{\cdot}H_2O$; (**b**) GCD curve

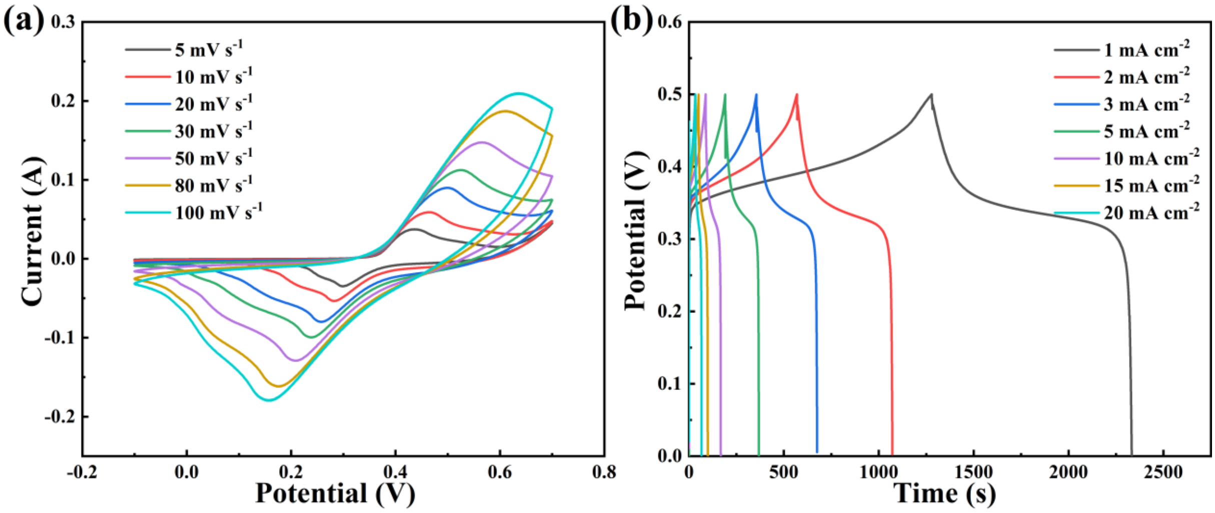

Figure S3. (**a**) CV curve of P−$MnMoO_4$; (**b**) GCD curve

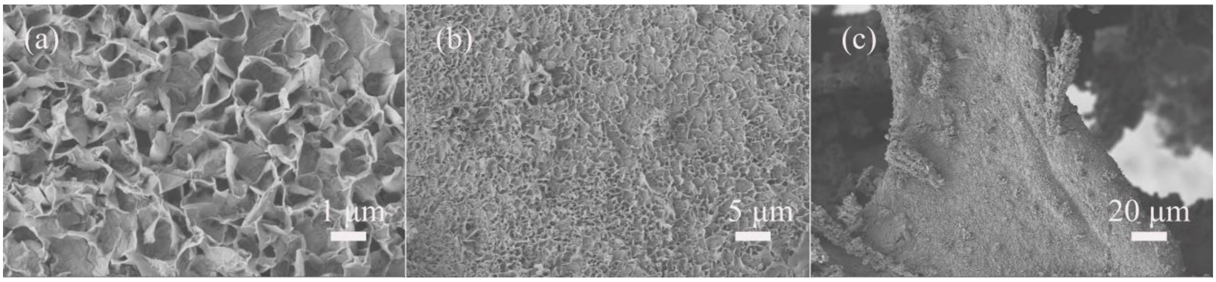

Figure S4. (**a−c**) SEM images of P−$MnMoO_4$ electrode material after charge/discharge cycle

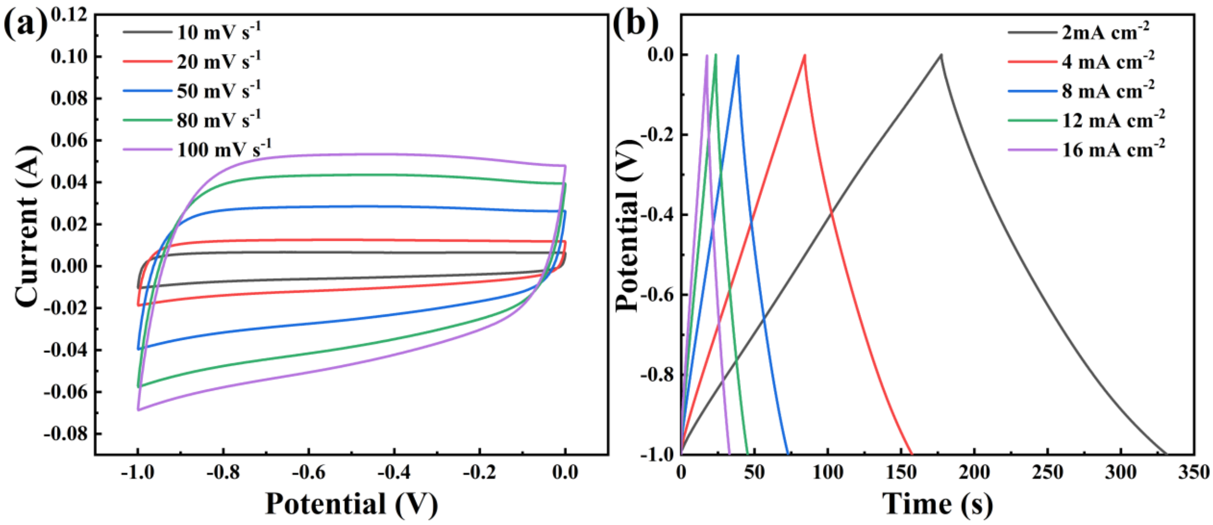

Figure S5. (**a**) CV curve of activated carbon; (**b**) GCD curve

Table S1. Performance comparison of hybrid supercapacitor based on $MnMoO_4$ electrode material with other reported devices.

Electrode material	Electrolyte	Specific capacitance	Energy density	Power density	Cyclic life	References
$MnMoO_4 \cdot nH_2O$	1 M NaOH	1271 F g^{-1}at 5 mV s^{-1}	31.6 Wh kg^{-1}	935 W kg^{-1}	84.5%,2000	[1]
$MnMoO_4$/NF	KOH/PVA	0.429 F cm^{-2}at 2 mA cm^{-2}	0.41 mWh cm^{-3}	0.41 mWh cm^{-3}	71.4%,10000	[2]
α−$MnMoO_4$ nanoparticles	2 M NaOH	200 F g^{-1}at 1.6 A g^{-1}	11 Wh kg^{-1}	100 W kg^{-1}	89.1%,1000	[3]
su−GC@ $MnMoO_4$	2 M KOH	528 F g^{-1}at 2 A g^{-1}	35.4 Wh kg^{-1}	223 W kg^{-1}	98.7%,5000	[4]
α−$MnMoO_4$/PANI	1 M Na_2SO_4	396 F g^{-1}at 5 mV s^{-1}			81%,500	[5]
$MnMoO_4$@MWCNT	1 M KOH	1017 F g^{-1}at 1 A g^{-1}	18.1 Wh kg^{-1}	362.4 W kg^{-1}		[6]
$MnMoO_4$/$MnCO_3$	2 M NaOH	1311 F g^{-1}at 1 A g^{-1}	116.8 Wh kg^{-1}	383 W kg^{-1}	85%,2000	[7]
$MnMoO_4$/$NiWO_4$	2 M KOH	598 F g^{-1}at 1 A g^{-1}			82%,5000	[8]
This Work (P−$MnMoO_4$)	2 M KOH	2.112 F cm^{-2}at1 mA cm^{-2} 1760 F g^{-1}at0.83 A g^{-1}	41.9 Wh kg^{-1}	666.8 W kg^{-1}	84.5%,10000	

References

1. Mu, X.; Zhang, Y.; Wang, H.; Huang, B.; Sun, P.; Chen, T.; Zhou, J.; Xie, E.; Zhang, Z., A high energy density asymmetric supercapacitor from ultrathin manganese molybdate nanosheets. *Electrochimica Acta* **2016,** 211, 217-224.
2. Prabakaran, P.; Arumugam, G.; Ramu, P.; Selvaraj, M.; Assiri, M. A.; Rokhum, S. L.; Arjunan, S.; Rajendran, R., Construction of hierarchical MnMoO4 nanostructures on Ni foam for high-performance asymmetric supercapacitors. *Surfaces and Interfaces* **2023,** 40.
3. Senthilkumar, B.; Selvan, R. K.; Meyrick, D.; Minakshi, M., Synthesis and Characterization of Manganese Molybdate for Symmetric Capacitor Applications. *International Journal of Electrochemical Science* **2015,** 10, (1), 185-193.
4. Appiagyei, A. B.; Asiedua-Ahenkorah, L.; Bathula, C.; Kim, H.-S.; Han, S. S.; Rao, K. M.; Anang, D. A., Rational design of sucrose-derived graphitic carbon coated MnMoO4 for high performance asymmetric supercapacitor. *Journal of Energy Storage* **2023,** 58.
5. Yesuraj, J.; Elumalai, V.; Bhagavathiachari, M.; Samuel, A. S.; Elaiyappillai, E.; Johnson, P. M., A facile sonochemical assisted synthesis of α-MnMoO 4 /PANI nanocomposite electrode for supercapacitor applications. *Journal of Electroanalytical Chemistry* **2017,** 797, 78-88.
6. Bhagwan, J.; Hussain, S. K.; Krishna, B. V.; Yu, J. S., Facile synthesis of MnMoO4@ MWCNT and their electrochemical performance in aqueous asymmetric supercapacitor. *Journal of Alloys and Compounds* **2021,** 856, 157874.
7. Pallavolu, M. R.; Banerjee, A. N.; Nallapureddy, R. R.; Joo, S. W., Urea-assisted hydrothermal synthesis of MnMoO4/MnCO3 hybrid electrochemical electrode and fabrication of high-performance asymmetric supercapacitor. *Journal of Materials Science & Technology* **2022,** 96, 332-344.
8. Feng, X.; Huang, Y.; Chen, M.; Chen, X.; Li, C.; Zhou, S.; Gao, X., Self-assembly of 3D hierarchical MnMoO4/NiWO4 microspheres for high-performance supercapacitor. *Journal of Alloys and Compounds* **2018,** 763, 801-807.
